# Characterization of nontyphoidal *Salmonella* strains from a tertiary hospital in China: serotype diversity, multidrug resistance, and genetic insights

**DOI:** 10.3389/fcimb.2023.1327092

**Published:** 2024-01-09

**Authors:** Wanshan Ma, Xiaodi Cui, Xiutao Dong, Xinpeng Li, Ke Liu, Yujiao Wang, Xiaohong Shi, Liang Chen, Mingju Hao

**Affiliations:** ^1^ Department of Clinical Laboratory Medicine, The First Affiliated Hospital of Shandong First Medical University & Shandong Provincial Qianfoshan Hospital, Shandong Medicine and Health Key Laboratory of Laboratory Medicine, Jinan, China; ^2^ School of Clinical Medicine, Jining Medical University, Jining, China; ^3^ Department of Bacterial Infectious Disease Control and Prevention, Shandong Center for Disease Control and Prevention, Jinan, China; ^4^ Center for Discovery and Innovation, Hackensack Meridian Health, Nutley, NJ, United States; ^5^ Department of Medical Sciences, Hackensack Meridian School of Medicine, Nutley, NJ, United States

**Keywords:** nontyphoidal *Salmonella*, antibiotic resistance, *mrkABCDF* operon, phylogenetic analysis, biofilm

## Abstract

**Objective:**

Nontyphoidal *Salmonella* is a significant public health concern due to its ability to cause foodborne illnesses worldwide. This study aims to characterize the nontyphoidal *Salmonella* strains isolated from patients in China.

**Methods:**

A total of 19 nontyphoidal *Salmonella* strains were characterized through serovar identification, antimicrobial susceptibility testing (AST), biofilm formation assessment. Genetic relatedness was determined using pulsed-field gel electrophoresis (PFGE). WGS was employed to decipher the resistance mechanism and to contextualize the *S.* serovar Mbandaka strains among previously sequenced isolates in China. The biofilm associated *mrkA* gene was examined by PCR.

**Results:**

The predominant serovar identified was *S.* Enteritidis, followed by *S.* Mbandaka, *S.* Thompson, *S.* Livingston, *S.* Alachua, and *S.* Infantis. PFGE analysis indicated a notable genetic similarity among the *S.* Mbandaka isolates. Phylogenetic analysis suggested that these strains were likely derived from a single source that had persisted in China for over five years. One multidrug resistance (MDR) *S.* Enteritidis isolate carried a highly transferable IncB/O/K/Z plasmid with *bla*_CTX-M-15_. One *S.* Thompson strain, harboring the *mrkABCDF* operon in an IncX1 plasmid, isolated from cutaneous lesions, demonstrated robust biofilm formation. However, no *mrkABCDF* loci were detected in other strains.

**Conclusion:**

Our study emphasizes the importance of persisted surveillance and prompt response to *Salmonella* infections to protect public health. The dissemination of *bla*_CTX-M-15_-harboring IncB/O/K/Z plasmid and the spread of virulent *mrkABCDF* operon among *Salmonella* in China and other global regions warrant close monitoring.

## Introduction

Nontyphoidal *Salmonella* (NTS) is one of the most common agents of gastrointestinal disease globally (Amuasi and May, 2019). It is estimated that NTS cause about 129.5 million cases each year, resulting in about 100,000 to 1 million deaths per year worldwide (Wen et al., 2017; Branchu et al., 2018). Salmonella infections frequently occur in humans when they consume contaminated foods, including poultry, eggs, beef, pork, milk, seafood, and fresh fish products. Additionally, direct interactions with animals can lead to the transmission of Salmonella to humans (Zhao et al., 2003). While most infections result in self-limiting gastroenteritis, individuals such as infants, the elderly or those with compromised immune systems are susceptible to life-threatening invasive infections (Marchello et al., 2022).

To date, over 2600 *Salmonella* serovars have been identified, of which only a few NTS serovars are responsible for most human infections (Akinyemi et al., 2023). *S.* Typhimurium and *S.* Enteritidis accounts for the majority of NTS infections in humans (Wang et al., 2023). Alarmingly, the emergence of multi-drug-resistant (MDR) *Salmonella* serovars, particularly against 3rd-generation cephalosporins and fluoroquinolones, is having a great impact on the efficacy of antibiotic treatment, and an increasing prevalence of MDR strains may lead to an increase in mortality rates of *Salmonella* infections (Crump et al., 2015). Of note, MDR strains can carry specific virulence factors which are more virulent than their susceptible counterparts (Gebreyes et al., 2009).

In China, surveillance data spanning from 2006 to 2019 reveals an average of 62 serovars are detected annually from human origin (Wang et al., 2023). *S.* Typhimurium and *S.* Enteritidis were the most common serovar causing human infections in China. Moreover, the proportion of antimicrobial-resistant *Salmonella* isolates occur with increasing frequency during 2006–2019, especially beta-lactam, quinolone, tetracycline, and rifampicin resistance (Wang et al., 2023). These findings highlight the need for continuous monitoring of *Salmonella* infection with particular emphasis on MDR *Salmonella* strains.

In this study, we identified and examined a total of 19 NTS isolates from patients at a tertiary hospital in eastern China in 2022. The majority of cases were reported between July and October, with the peak incidence occurring in October. Through a combination of phenotypic and molecular analyses, we aimed to gain comprehensive insights into the characteristics of these *Salmonella* strains.

## Materials and methods

### Bacterial isolates

**Figure 1:**
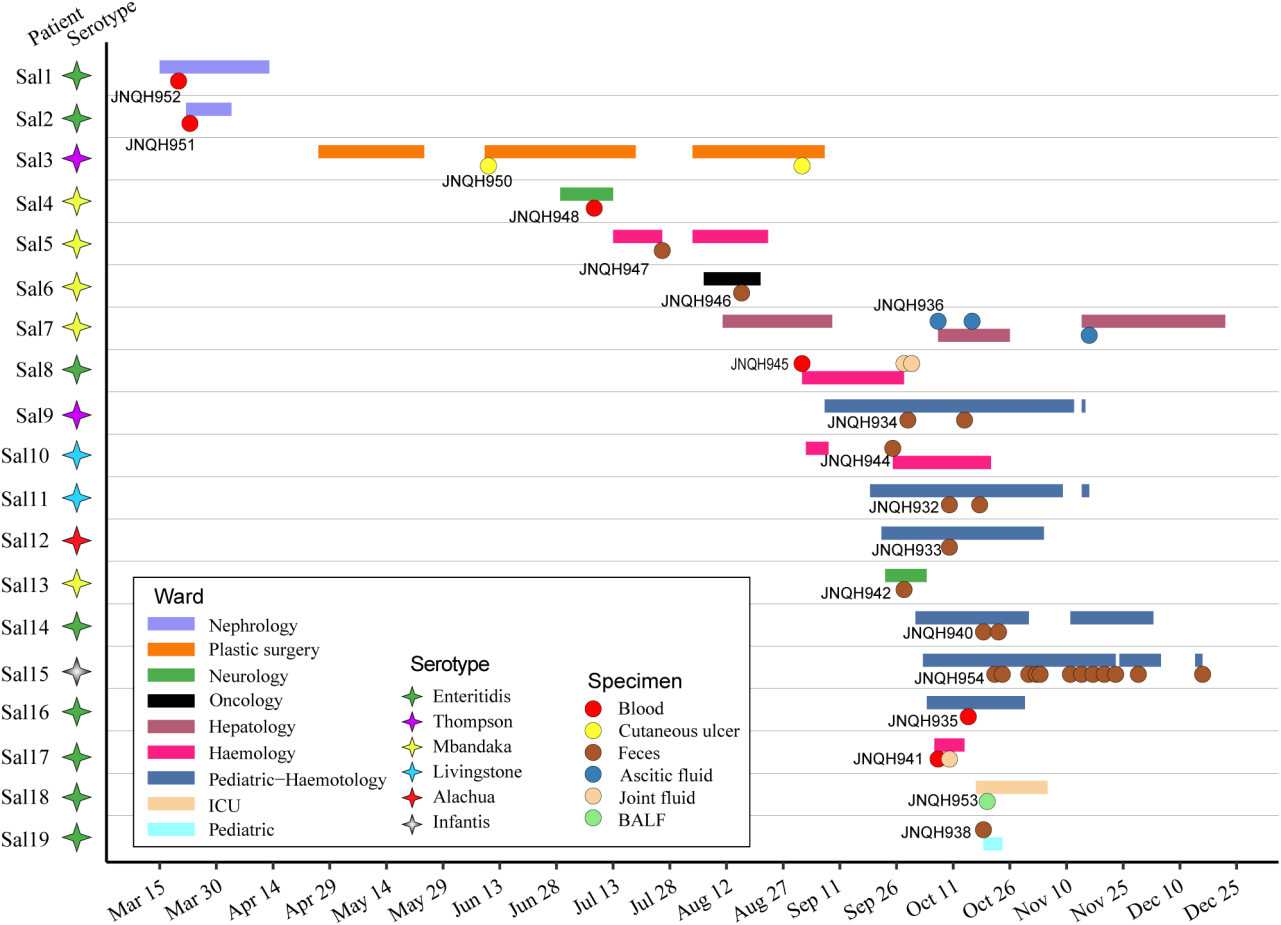
Time frame of stays in hospitalization for the 19 patients involved in NTS infection. Horizontal bars represent the duration of hospitalization for each patient, with distinct colors indicating the respective ward departments. Circles represent the time of isolation for each patient with colored circles representing the specimen source. Colored stars represent different serovars.

During the period from March 20, 2022, to December 16, 2022, a total of 19 isolates of NTS were investigated in this study (**Figure 1**). All strains were the first isolates collected from each patient. Ten of the isolates were obtained from feces. Other clinical specimens included blood cultures, bronchoalveolar lavage fluid, cutaneous ulcer, ascitic fluid, joint fluid. For routine use samples were inoculated on Blood Agar (TSA w/5% Sheep Blood) and selective SS Agar (Salmonella-Shigella agar) and were grown at 37°C overnight.

### Patient information

The median age of infected patients was 35.5 years (range: 1-78 years). 17 of the patients were males. The median duration of illness was 9 days (range: 5-89 days). Predominant symptoms included diarrhea (68.4%), fever (47.4%), abdominal pain (47.4%). Lower respiratory tract infection-related symptoms and signs are prominent in a patient with lung infection (patient Sal18). All the patients were administered antimicrobial agents, with 14 (73.7%) patients showing effective outcomes. Short descriptions of the investigated isolates are presented in **Table 1**.

**Table 1:** Overall strain features collected in this study.

Patient ID	Strain	Gender	Age (Year)	Specimen type	Serovar	Ward department	Main Diagnosis
Sal1	JNQH952	Male	25	Blood	Enteritidis	nephrology	Post-renal transplantation
Sal2	JNQH951	Male	40	Blood	Enteritidis	nephrology	Systemic vasculitis
Sal3	JNQH950	Male	58	Skin secretion	Thompson	Plastic surgery	Diabetes mellitus
Sal4	JNQH948	Male	78	Blood	Mbandaka	Neurology	Encephalitis
Sal5	JNQH947	Male	67	Feces	Mbandaka	Haemology	Lymphoma
Sal6	JNQH946	Male	58	Feces	Mbandaka	Oncology	Cholangiocarcinoma
Sal7	JNQH936	Male	39	Ascites	Mbandaka	Hepatology	Acute pancreatitis
Sal8	JNQH945	Male	40	Blood/Joint fluid	Enteritidis	Haemology	Acute lymphocytic leukemia
Sal9	JNQH934	Male	1	Feces	Thompson	Pediatric-Haemotology	Acute myeloid leukemia
Sal10	JNQH944	Female	39	Feces	Livingstone	Haemology	Aplastic anemia
Sal11	JNQH932	Male	10	Feces	Livingstone	Pediatric-Haemotology	Aplastic anemia
Sal12	JNQH933	Male	12	Feces	Alachua	Pediatric-Haemotology	Aplastic anemia
Sal13	JNQH942	Male	71	Feces	Mbandaka	Neurology	Cerebral infarction
Sal14	JNQH940	Male	10	Feces	Enteritidis	Pediatric-Haemotology	Hematopoietic stem cell transplantation
Sal15	JNQH954	Female	3	Feces	Infantis	Pediatric-Haemotology	Acute lymphocytic leukemia
Sal16	JNQH935	Male	11	Blood	Enteritidis	Pediatric-Haemotology	Acute lymphocytic leukemia
Sal17	JNQH941	Male	32	Blood	Enteritidis	Haemology	Thrombocytopenia
Sal18	JNQH953	Male	65	BALF	Enteritidis	ICU	Pneumoniae
Sal19	JNQH938	Male	5	Feces	Enteritidis	Pediatric	Gastroenteritis

### Antimicrobial susceptibility testing

AST was performed using the disk diffusion method or microdilution method as recommended by the Clinical and Laboratory Standards Institute guidelines (CLSI, 2021). Antimicrobial discs were manufactured by Oxoid in UK. The following antimicrobial agents were tested: ampicillin (AMP, disk content 10μg), ceftazidime (CAZ, 30μg), ciprofloxacin (CIP, 5μg), ceftriaxone (CRO, 30μg), trimethoprim-sulfamethoxazole (TMP-SMZ, 1.25/23.75μg), meropenem (MEM, 10μg). Levofloxacin (LEV) was tested by microdilution method. Results were interpreted using the CLSI breakpoints (CLSI M100, 2021). MDR was defined as acquired non-susceptibility to at least one agent in three or more antimicrobial categories (Magiorakos et al., 2012). Each of the antibiotics was tested with 3 duplicates. ATCC 25922 (*E. coli*) were used as quality control strains for susceptibility testing.

### Salmonella serotyping

The isolated *Salmonella* were serotyped on slides by slide agglutination as described in the manufacturer’s instructions (Statens Serum Institut, Copenhagen, Denmark). Briefly, the *Salmonella* strains were grown overnight on Blood Agar then colonies were transferred to the drop of antiserum. The “O” antigen type was determined based on oligosaccharides associated with lipopolysaccharide. Then the “H” antigen was determined based on flagellar proteins. The reaction was read with the naked eye by holding the slide in front of a light source against a black background. A positive reaction was seen as a visible agglutination. A negative reaction was persistence of the homogeneous milky turbidity. The serovar was assigned according to the Kauffmann-White scheme, which is a comprehensive system employed for the discrimination of serological variations within the *Salmonella* genus.

### Pulsed-field gel electrophoresis

All isolates were analyzed for genetic relatedness by PFGE. PFGE of Xbal-digested genomic DNA samples were performed with a CHEF MAPPER XA apparatus (Bio-Rad, USA), as previously described (Ma et al., 2023). In brief, genomic DNA was prepared by embedding cells in agarose plugs, followed by XbaI digestion for 2 h at 37°C. Electrophoresis conditions consisted of one phase from 2.2 to 63.8 s at a run time of 17 h. *Salmonella* serovar Branderup H9812 strain was used as the reference strain. PFGE patterns were analyzed and compared using GelJ software 2.0v (Heras et al., 2015).

### Biofilm formation

The process of biofilm formation was assessed using crystal violet staining as described previously with minor modifications (Hao et al., 2021a). Briefly, overnight cultures of the bacteria were diluted 1:1,000 in LB medium. A total volume of 200 μl of the diluted culture was then transferred to each well of a microtiter plate. The plate was incubated at 37°C for 24 hours. After incubation, the wells were washed four times with distilled water to remove any planktonic (free-floating) bacteria. The remaining adherent bacteria were then stained with 125 μl of a 0.1% crystal violet dye solution. Following incubation for 10 minutes, the crystal violet solution was carefully removed, and the wells were washed six times with distilled water. After that, 150 μl of a 30% glacial acetic acid in water solution was added to each well. The plate was incubated for an additional 10 minutes at room temperature, allowing the acetic acid to dissolve the crystal violet-stained biofilms. The optical density (OD) of the solubilized biofilms was then measured at a wavelength of 590 nm using a microplate reader (Thermo Scientific). At least three replicates were performed for each sample. Statistical significance in biofilm assays was calculated using Student’s t-test and any differences between strains with a *P* value of <0.05 were considered significant.

### Whole-genome sequencing

Complete sequencing was performed on four selected strains, which included a strong biofilm-forming *S.* Thompson strain (JNQH950), *S.* Mbandaka (JNQH948), as well as two resistant *S.* Enteritidis strains (JNQH940 and JNQH952). Genomic DNA was isolated using a WizardR Genomic DNA Purification Kit as described in the manufacturer’s instructions (Promega, Madison, WI, USA). The DNA samples were subject to next generation sequencing using the Illumina HiSeq (Illumina, San Diego, CA, USA) and Oxford Nanopore (MinION system). The hybrid assembly was performed by Unicycler v0·5.0 (Wick et al., 2017). The whole-genome sequences were annotated by Prokka (Seemann, 2014) automatically followed by manual curation.

### Genomic analysis

*In silico* multi-locus sequence typing was performed using MLST 2.0 (Larsen et al., 2012). Seven housekeeping genes, including *aroC, dnaN, hemD, hisD, purE, sucA*, and *thrA*, were chosen for MLST analysis. The antibiotic resistome(s) were predicted by the Resistance Gene Identifier (RGI) (https://github.com/arpcard/rgi) to query the CARD database (https://card.mcmaster.ca) (Alcock et al., 2020). The virulence genes and plasmid replicons in the sequenced isolates were identified using VFDB (Liu et al., 2022) and PlasmidFinder 2.0 (Carattoli et al., 2014) respectively. In order to contextualize our isolates among the corresponding strains in China, genomes of *S.* Mbandaka were downloaded from EnteroBase in combination with the genomes reported in a large-scale one-health study (Wang et al., 2023). SNPs were detected using Snippy v3.2 (https://github.com/tseemann/snippy) based on recombination filtration by Gubbins v. 2.4.1 (Croucher et al., 2015) and core SNP extraction by SNP-sites v. 2.5.1 (Page et al., 2016). SNPs located in phage regions, repetitive and recombinogenic regions, were removed before phylogenetic analysis. Genomic coordinates corresponding to phage sequences and repetitive elements in the reference genome were determined using Phast (http://phaster.ca) and Mummer (Kurtz et al., 2004), respectively. All extracted SNPs in core genome regions were concatenated as pseudosequences. The approximately-maximum-likelihood phylogenetic tree was inferred from alignments of nucleotide sequences with FastTree (Price et al., 2009). The visualization and annotation of the phylogenetic tree were carried out using R ggtree package (Yu et al., 2017).

OriT Finder was used to determine the conjugation module (Li et al., 2018). ISFinder was used to identify ISs (https://isfinder.biotoul.fr/). Plasmid similarity was examined comparing against the PLSDB plasmid database (Schmartz et al., 2022). Comparison between homologous plasmids were performed using BLASTn and illustrated by Easyfig v2.2.2 (Sullivan et al., 2011). To examine the distribution and features of *mrkABCDF* operon in global plasmids, plasmid reference sequences were downloaded from NCBI (https://ftp.ncbi.nlm.nih.gov/refseq/release/plasmid) and compared using Mashtree (Katz et al., 2019). Serovars assigned from Kauffmann-White scheme were confirmed by SeqSero2 v1.1.1 (Zhang et al., 2019).

### Conjugal transference of bla_CTX-M-15_ harboring plasmid

In order to examine the conjugal transference of *bla*_CTX-M-15_ harboring plasmid in JNQH940, conjugation experiments were conducted using *E. coli* J53AziR as recipients, following a previously described method (Hao et al., 2021b). Overnight cultures of JNQH940 and *E. coli* J53AziR were mixed in a 1:1 ratio and applied separately onto 0.45-μm filter paper. The filter papers were then placed on LB agar plates and incubated overnight at 37°C. Transconjugants were selected on Mueller–Hinton agar containing sodium azide (100 mg/L) and ceftazidime (16 mg/L). The presence of transconjugants was confirmed using PCR targeting the *bla*_CTX-M-15_ gene. Conjugation frequency was determined by dividing the number of transconjugants by the number of recipient cells.

### Detection of m*rkABCDF* loci by PCR

The *mrkABCDF* virulence loci were identified via a colony Polymerase Chain Reaction (PCR) assay, wherein the target gene *mrkA* was amplified using specific primers (mrkAF: 5’-ACCAGCAAACAACAGGGCTA-3’, mrkAR: 5’-TGATTTTGTTGGTCAGCGCG-3’). The occurrence of a positive outcome in this assay was determined by the generation of a DNA fragment with a length of 106 base pairs. For the purpose of assay validation, a well-characterized *K. variicola* isolate JNQH473, which had previously undergone comprehensive sequencing in our prior research (Wang et al., 2021a), was utilized as a positive control.

### Nucleotide sequence accession numbers

Complete sequences of the chromosomes and plasmids of strain JNQH940, 948, 950 and 952 have been deposited in the GenBank databases under accession numbers CP136141-CP136151.

## Results

### Serovar identification and antimicrobial susceptibility

Six different serovars were assigned according to the Kauffmann-White scheme. *S.* Enteritidis was the most common serovar, accounting for 42.1% of the isolates, followed by *S.* Mbandaka (26.3%), Thompson (10.5%), Livingston (10.5%), Alachua (5.3%), Infantis (5.3%) (**Figure 1**). Antimicrobial-susceptibility testing results showed that isolates of *S.* Mbandaka, Infantis, Livingston, and Alachua exhibited susceptibility to the tested antibiotics. In contrast, JNQH934 (*S.* Thompson) and JNQH940 (*S.* Enteritidis) demonstrated significant resistance to AMP, CIP, and TMP-SMZ, displaying multidrug resistance (**Table 2**).

**Table 2:** Antimicrobial susceptibility testing for NTS isolates collected from this study.

No. Strains (Serovar)	Antibiotic agents						
	AMP^a^	CAZ^a^	CIP^a^	CRO^a^	LEV^b^	TMP-SMZ^a^	MEM^a^
JNQH933 (Alachua)	22	30	35	32	0.125	30	29
JNQH952 (Enteritidis)	6	22	34	32	0.06	22	30
JNQH953 (Enteritidis)	6	27	31	27	0.125	16	31
JNQH935 (Enteritidis)	6	24	38	30	1	20	31
JNQH941 (Enteritidis)	23	27	35	30	1	20	35
JNQH945 (Enteritidis)	6	25	31	30	0.25	20	35
JNQH940 (Enteritidis)	6	6	21	6	2	6	30
JNQH938 (Enteritidis)	6	25	27	30	0.25	17	32
JNQH951 (Enteritidis)	6	25	31	30	0.03	6	38
JNQH954 (Infantis)	24	29	30	29	0.125	30	32
JNQH944 (Livingstone)	19	25	36	27	0.125	26	30
JNQH932 (Livingstone)	19	27	36	29	0.125	27	31
JNQH946 (Mbandaka)	22	22	34	30	0.06	27	33
JNQH948 (Mbandaka)	23	26	38	30	0.125	24	31
JNQH936 (Mbandaka)	23	24	39	30	0.125	20	30
JNQH947 (Mbandaka)	22	25	35	30	0.125	27	30
JNQH942 (Mbandaka)	20	21	35	30	0.125	25	30
JNQH950 (Thompson)	6	28	31	34	0.125	6	36
JNQH934 (Thompson)	6	22	6	30	1	6	32

^a^Disk infusion method (mm); ^b^Microdilution method (μg/ml). AMP, ampicillin; CAZ, ceftazidime; CIP, ciprofloxacin; CRO, ceftriaxone; LEV, levofloxacin; TMP-SMZ, trimethoprim-sulfamethoxazole; MEM, meropenem. All tests were performed in duplicate, and each test included three biological replicates.

### Genetic relatedness of *Salmonella* isolates

**Figure 2:**
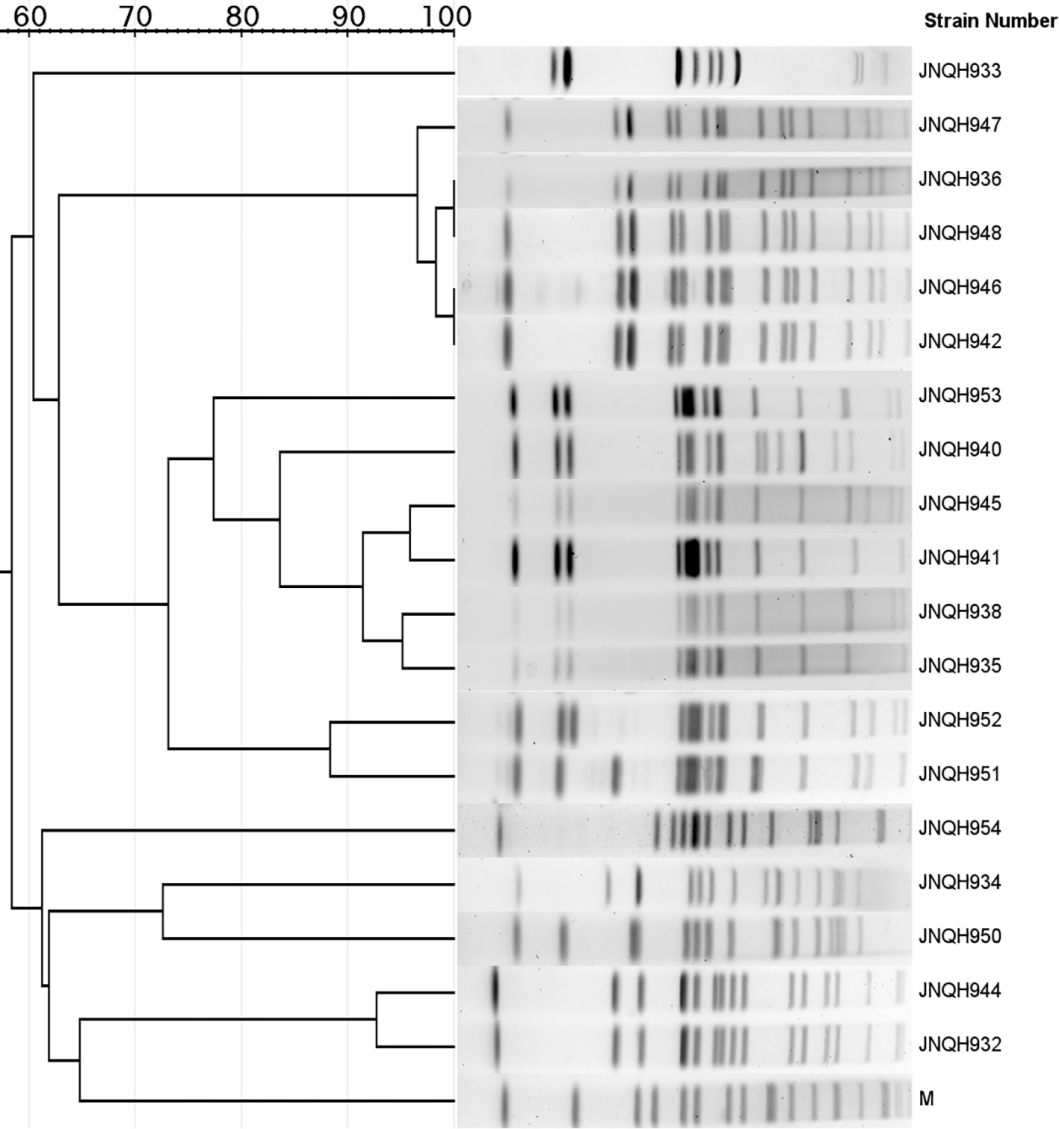
Dendrogram of the pulsed-field gel electrophoresis (PFGE) patterns of NTS isolates collected from this study. M, PFGE marker strain (*S.* Braenderup H9812).

**Figure 3:**
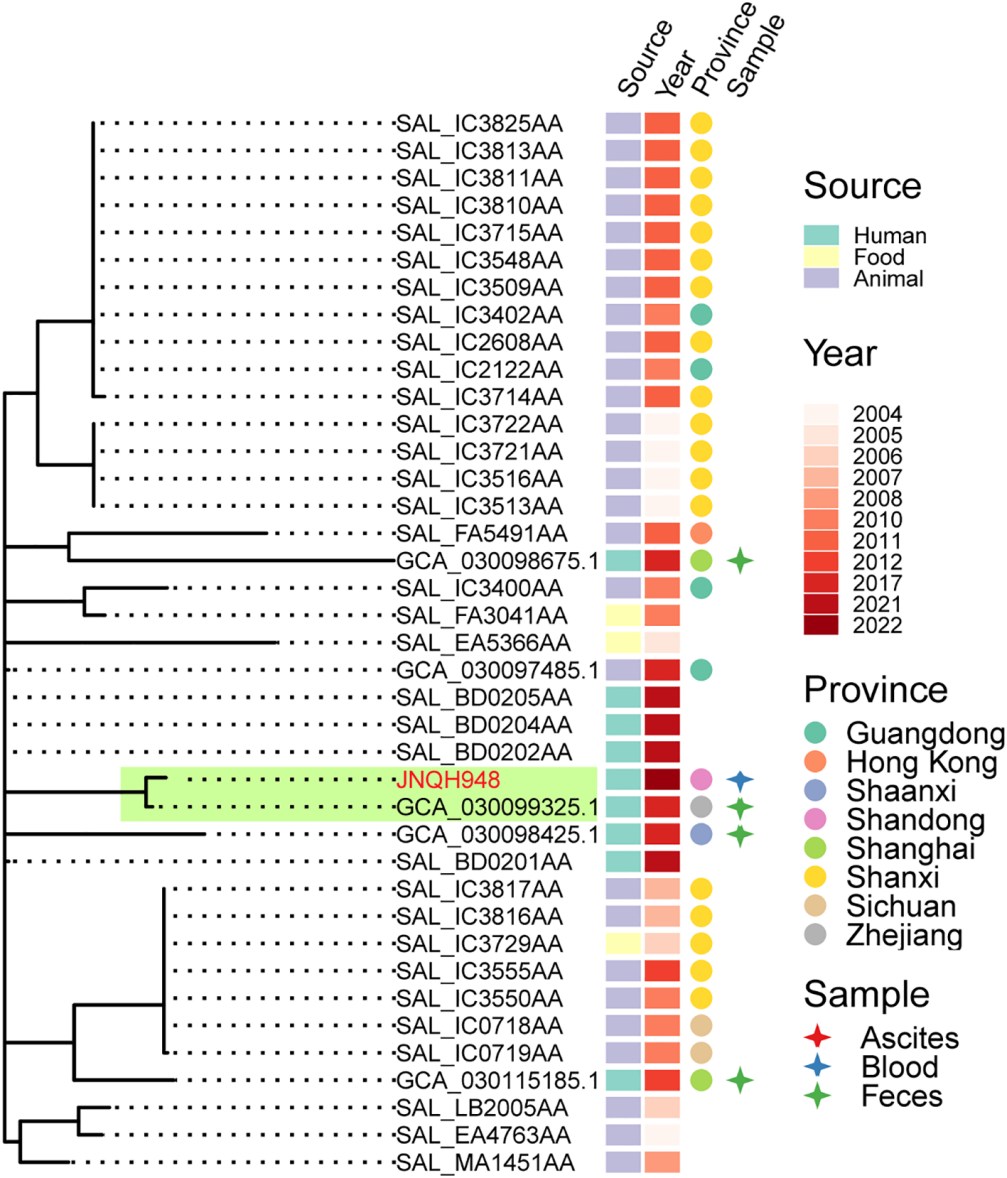
Phylogenetic relationships within *S.* Mbandaka strain JNQH948 and publicly available sequences from EnteroBase and a Chinese study. Phylogeny (left) showing the relationships. The strain source, collection year, location and specimen types were shown for each isolate. Subclade containing the JNQH948 and the closely related *S.* Mbandaka strain were indicated in light green shade.

PFGE analysis utilizing XbaI restriction enzyme was employed to evaluate the genetic relatedness among the isolates. The PFGE patterns of *S.* Enteritidis isolates were similar but distinguishable except JNQH941 and JNQH935. Two *S.* Thompson isolates (JNQH950, 934) had notably different PFGE patterns, indicative of genetic diversity. However, two *S.* Livingston isolates (JNQH932, 944) demonstrated similar PFGE patterns, suggesting a potential genetic relatedness between these isolates. Of note, the *S.* Mbandaka serovar isolates displayed very similar PFGE patterns, suggesting a closer genetic relationship (**Figure 2**). Interestingly, SNP-based phylogenetic tree uncovered a close relationship between *S.* Mbandaka JNQH948 and a strain (GCA_030099325.1) obtained from human feces in Zhejiang in 2017 (**Figure 3**), with only 8 SNP differences. These results highlight the occurrence of small-scale clonal spread of *S.* Mbandaka in China.

### Genomic and phenotypic characterization of *Salmonella* strains

Whole-genome sequences were obtained for four strains (JNQH948, 950, 952, 940). *S.* Enteritidis strains JNQH940 and JNQH952 harbored three and two plasmids respectively. *S.* Thompson strain JNQH950 carried two plasmids whereas no plasmids were identified in *S.* Mbandaka strain JNQH948. The serovars of all isolates predicted to be consistent with *Salmonella* serotyping by slide agglutination. MLST revealed the *S.* Mbandaka isolate (JNQH948) belonged to ST413. *S.* Enteritidis (JNQH940, 952) and S. Thompson (JNQH950) were classified as ST11 and ST26 respectively. The resistance genotypes predicted by RGI revealed ampicillin resistance were mediated by *bla*_TEM-1_. For the MDR JNQH940, resistance to quinolone (CIP) was conferred by *qnrS1*. The resistance of TMP-SMZ was associated with the presence of trimethoprim resistant *sul1/sul2* genes. JNQH940 was found to carry the *bla*_CTX-M-15_ gene. Notably, the virulence cluster of *mrkABCDF* was detected in *S.* Thompson strain JNQH950 but absent in other strains.

### bla_CTX-M-15_ was carried by a highly transferrable IncB/O/K/Z plasmid in *S.* Enteritidis strain JNQH940

**Figure 4:**
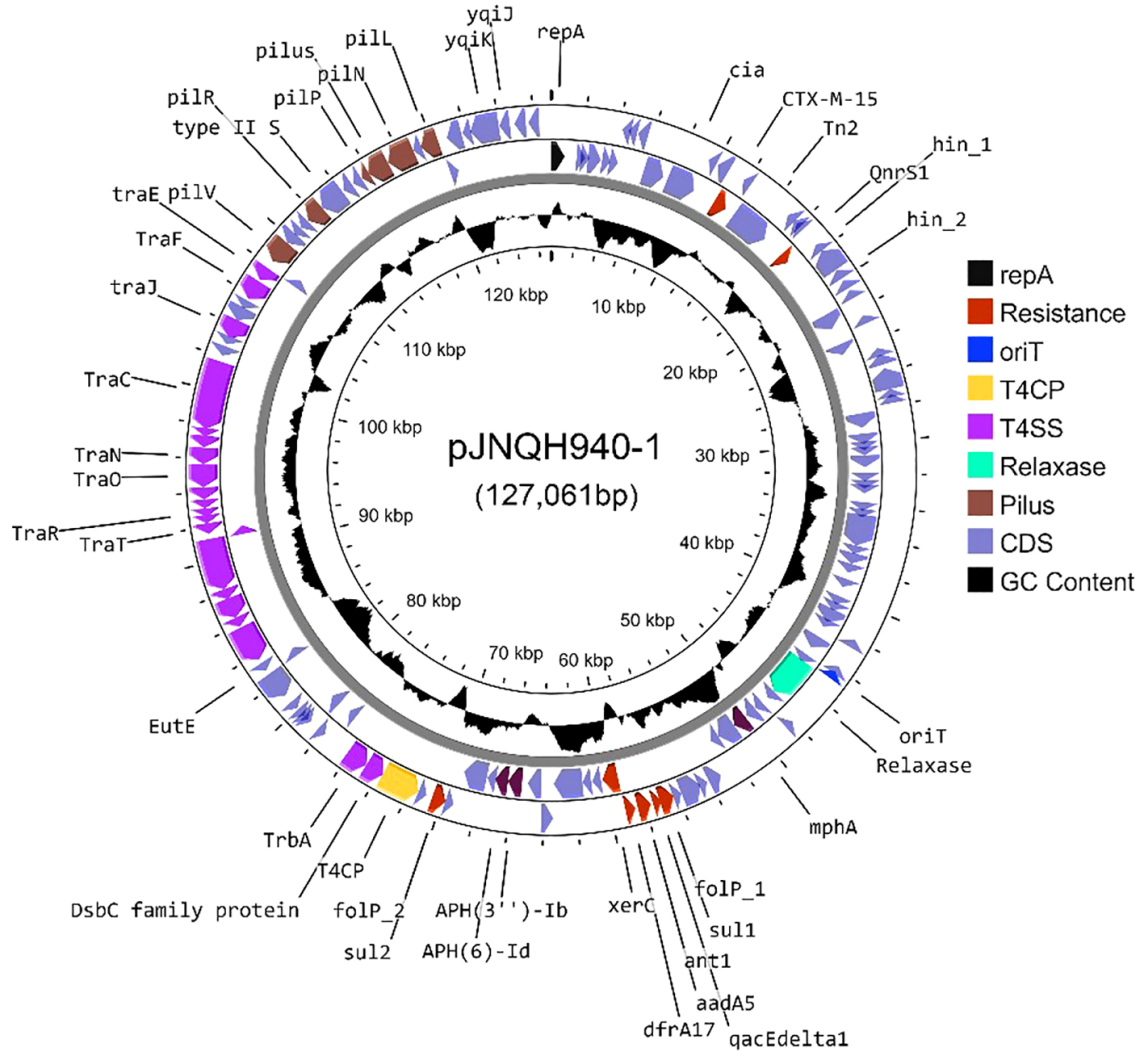
A circle map of the *bla*_CTX-M-15_-bearing IncB/O/K/Z type plasmid pJNQH940-1 in strain JNQH940. Open reading frames (ORFs) of pJNQH940-1 are shown as the outermost ring, with plasmid replicons, transconjugation elements (T4CP, T4SS, oriT, relaxase), type IV pilus system and antimicrobial resistance genes highlighted.

The *bla*_CTX-M-15_-harboring plasmid in strain JNQH940 was determined to be an IncB/O/K/Z plasmid type, with a length of 127,061bp base pairs. The plasmid contains genes associated with plasmid conjugation, as well as a region comprising genes to encode a type IV pilus system. The conjugation elements included the origin of transfer site (oriT), type IV coupling protein gene (T4CP), and genes encoding the relaxase and some type IV secretion components (T4SS) (**Figure 4**). Conjugation assays showed the IncB/O/K/Z plasmid was successfully transferred into *E. coli* J53 from JNQH940 strain. The conjugation frequency was 10^-3^ per recipient cell, which was further confirmed by PCR targeting the *bla*_CTX-M-15_ gene and IncB/O/K/Z replicon. In addition to *bla*_CTX-M-15_, the plasmid also harbored 9 additional antimicrobial resistance genes encoding resistance to fluroquinolone (*qnrS1*), macrolide (*mphA, mrx*), sulfonamide (*sul1/sul2*), antiseptics (*qacE*) and aminoglycoside [*aadA5, APH(3’)-Ib, APH(6)-Id*].

### JNQH950 exhibited significantly higher biofilm-forming capabilities mediated by *mrkABCDF* operon in an IncX1 plasmid

**Figure 5:**
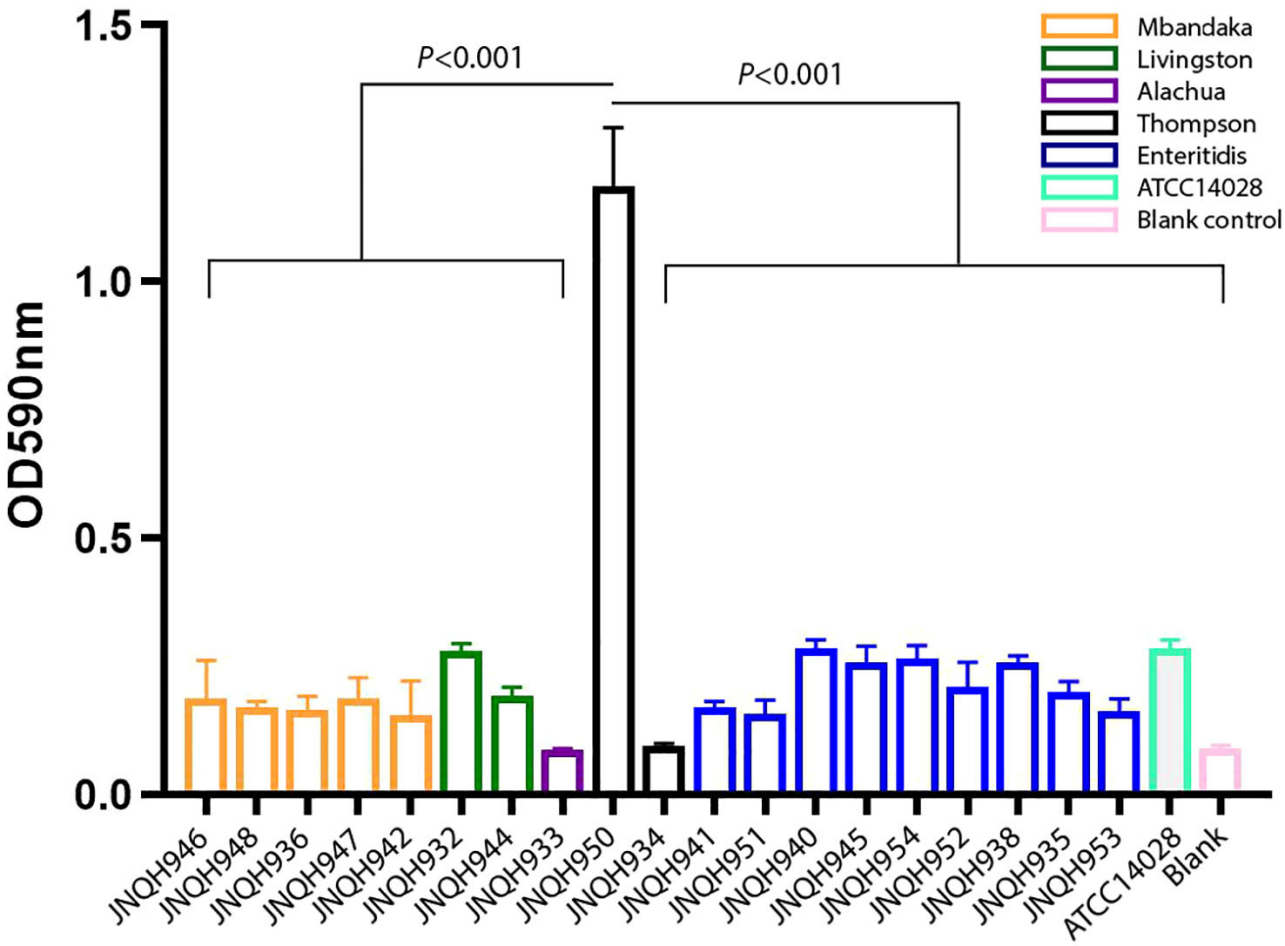
Biofilm formation of NTS strains. Data are represented as mean ± standard deviation from at least three independent experiments.

**Figure 6:**
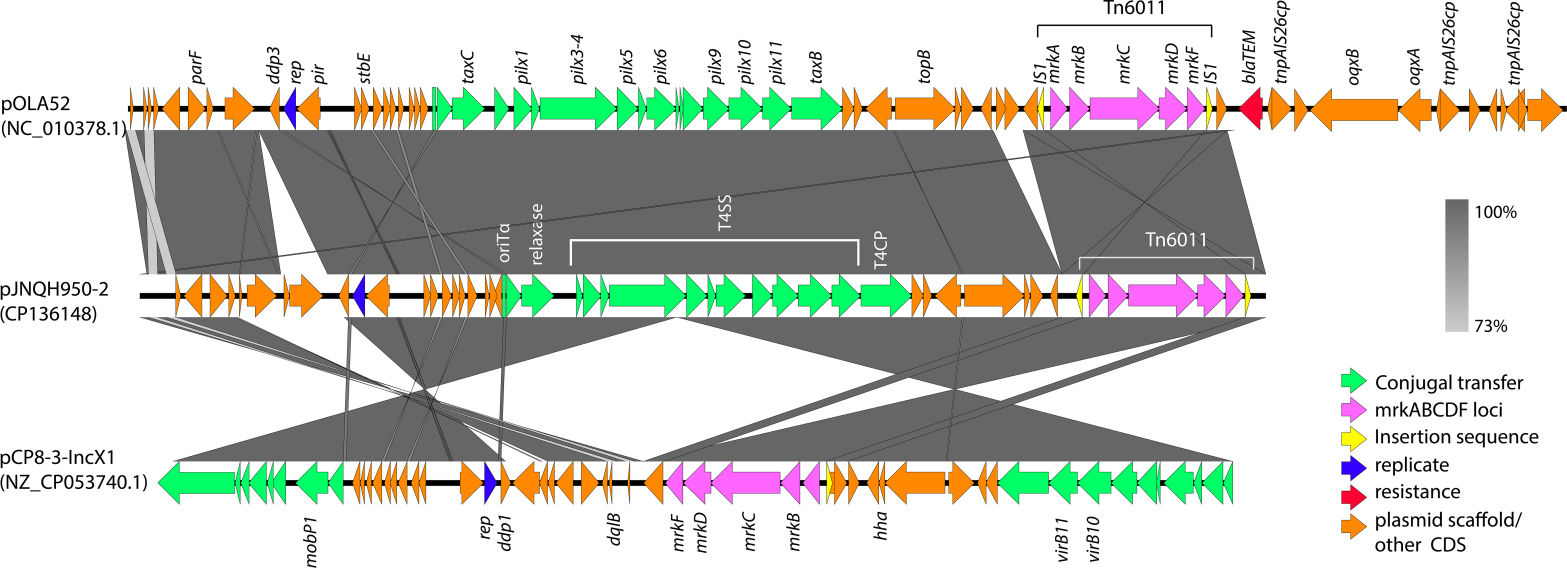
Genetic comparison of *mrkABCDF* harboring plasmid pJNQH950-2 and two closely related to plasmid pCP8-3-IncX1 (NZ_CP053740.1), pOLA52 (NC_010378.1). *mrkABCDF* transposon (Tn6011) are shown as the outermost ring. The genes are exhibited in arrows with different colors to note on their functional classes. Shades indicate shared regions of homology. The plasmid accession numbers are listed below the plasmid names.

We investigated the biofilm-forming capabilities of various *Salmonella* strains. The OD_590_ for JNQH950 was determined to be 1.187, while the other strains exhibited an OD_590_ measurement below 0.29. ATCC14028, which served as a reference, was measured to be 0.284. These results underscore the unique biofilm-forming potential of JNQH950 when compared to other tested strains (**Figure 5**). Further WGS analysis revealed the backbone structure of *mrkABCDF* cluster harboring plasmid belonged to an IncX1 plasmid, with a length of 40,394 base pairs. The *mrkABCDF* cassettes were contained within two IS1A elements of the IS1 family, constituting a transposon of Tn6011. These *mrkABCDF* cassettes encode the main structural subunit and assembly machinery of type 3 fimbriae, which are associated with biofilm formation. In addition, the MGEs associated with plasmid replication, stability, transfer integration was predicted. BLAST query against GenBank database showed that plasmid was closely related to plasmid pCP8-3-IncX1 (NZ_CP053740.1, with 90% coverage and 99.65% identity), pOLA52 (NC_010378.1, with 95% coverage and 99.47% identity), and some others plasmids hosted by *Enterobacterales* (**Figure 6**).

### Tracking key virulence loci encoding *mrkABCDF* operon in global plasmids revealed their prevalence in *Enterobacteriaceae*

**Figure 7:**
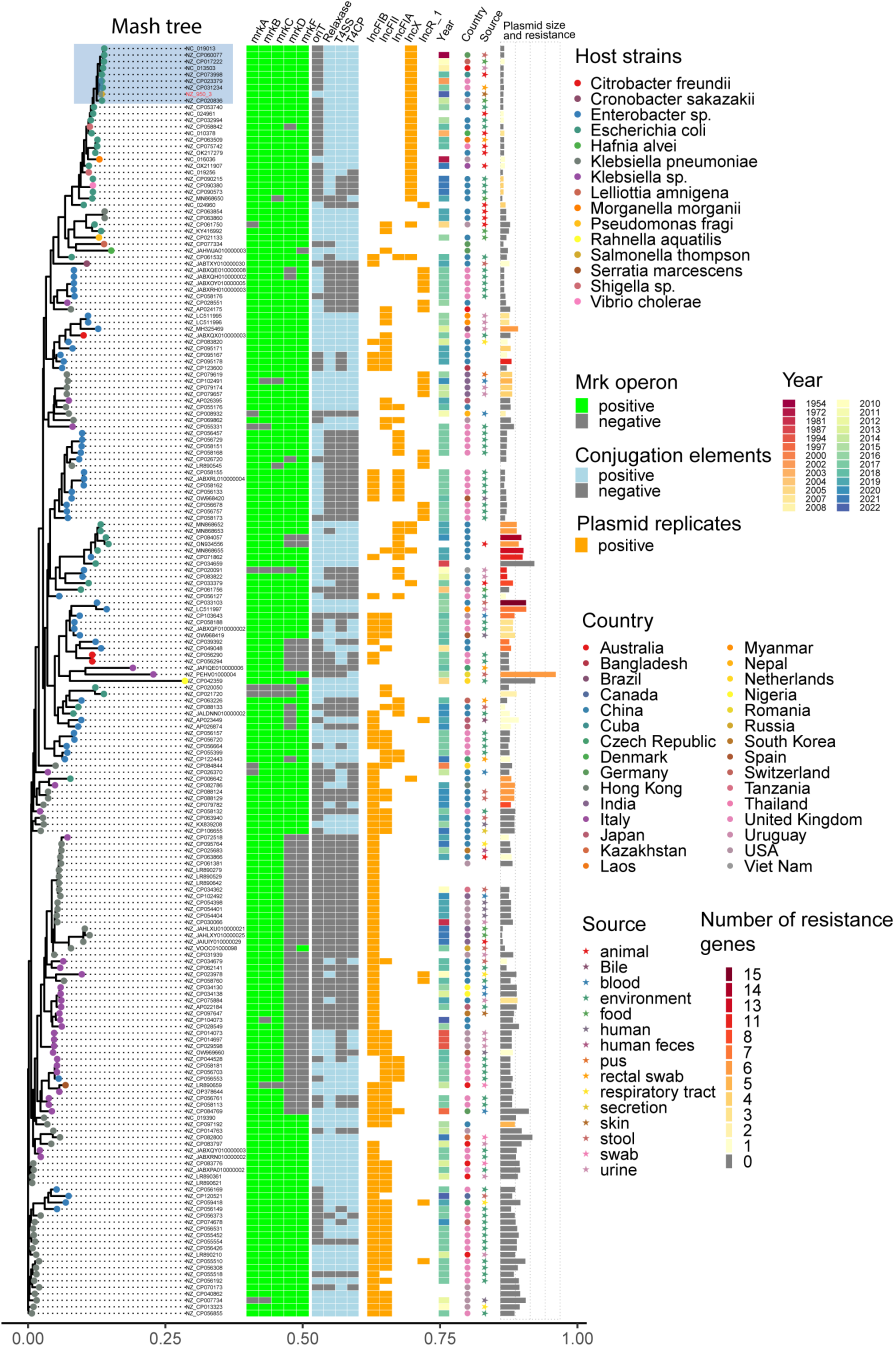
Distance tree of plasmids harboring *mrkABCDF* operon in reference NCBI database. Phylogeny (left) showing the relationships between 195 *mrkABCDF* operons harboring plasmids. The presence of *mrkABCDF* virulence loci, plasmid conjugation elements (oriT, T4SS, T4CP, relaxase), top five plasmid types, host strain properties (collection year, country, source), as well as the plasmid size and number of resistance genes were illustrated for each isolate. Grey tiles indicate gene’s absence. The isolates’ tip points are colored by the host species.

A total of 195 plasmids harboring *mrkABCDF* operon were identified in the reference plasmids in NCBI database, most of which are carried by *Enterobacteriaceae*. *K. pneumoniae* is the dominant host strain, followed by *E. coli*, *E. hormaechei, K. quasipneumoniae.* These plasmids belong to several incompatibility groups, predominant by IncFIB, IncFII, IncFIA, IncX, IncR. Plasmid sequence analysis by oriTfinder revealed the presence of four crucial conjugation elements (oriT, T4SS, T4CP, relaxase gene) in 31.2% (61/195) of the plasmids. The majority of these plasmids were found in host strains isolated in the environmental sources. The top 5 countries were UK, China, USA, India and Austria. Notably, plasmids originating from homo sapiens were most commonly detected in urine samples. It is noteworthy that 60 of the analyzed plasmids coharbored antibiotic resistance genes, including 6 carbapenem resistance genes and 17 *mcr* genes. *mrkABCDF* operon harboring plasmids has been present in *E. coli* strains dating back to the 1950s. Eight plasmids comprised of a cluster which had a close relationship with our plasmids, all of which belonged to IncX1 plasmid type and were carried by *E. coli* strains (**Figure 7**).

## Discussion

During the year 2022, our hospital diagnosed a total of 19 patients with *Salmonella* infections, as determined through microbiologic cultures. The most commonly identified serovar was *S.* Enteritidis, closely followed by *S.* Mbandaka and some other sporadic serovars including *S.* Thompson, Livingston, Alachua and Infantis. The majority of cases were reported between the months of July and October, with the highest peak occurring in October. Genetic relatedness analysis suggested that *S.* Mbandaka isolates were part of a single clonal expansion whereas *S.* Enteritidis strains were composed of diverse clonal linages. A significant observation was that *S.* Alachua was isolated from the feces of a patient suffering from a gastro-infection, marking the first observation of this particular serovar in China.

Non-typhoidal *Salmonella* infections typically manifest as mild and self-limiting acute enterocolitis in the majority of individuals (Crump et al., 2015). Parenteral infections, resulting from the dissemination of the bacterium beyond the gastrointestinal tract, are infrequent complications of gastroenteritis (Graham et al., 2000). However, reports of nontyphoidal *Salmonella* causing intravascular, bone, and joint infections are widespread on a global scale. In Africa, NTS strains cause invasive disease with bacteraemia more often in children, with 4100 deaths per year (Crump et al., 2015; Akinyemi et al., 2023). Such invasive infections are particularly associated with various forms of immunocompromise, with individuals at the extremes of age or those with compromised immune systems (Crump et al., 2011; Parry et al., 2013; Crump et al., 2015; Akinyemi et al., 2021). In our study, nine of our patients experienced extra-intestinal infections, underscoring the potential severity of the encountered *Salmonella* strains. Our cases demonstrated a heightened vulnerability to these infections, possibly due to the presence of multiple comorbidities, including acute leukemia, diabetes, autoimmune diseases, and organ transplantation. Given the impaired immunity in these patients, swift and comprehensive clinical intervention is essential. This is because *Salmonella* can penetrate the compromised intestinal barrier and disseminate to multiple organs, potentially leading to sepsis and severe complications. Therefore, proactive and diligent medical management is crucial for these individuals to minimize adverse outcomes.

Cutaneous infections caused by *Salmonella* are exceedingly rare occurrences. Only a few reports have documented cutaneous manifestations in patients with *S. enterica* infection (Marzano et al., 2003; Desikan et al., 2009; Wiegand et al., 2009; Alfouzan et al., 2017). In our study, we observed that *S.* Thompson strain JNQH950 isolated from the skin secretion displayed a remarkable capacity for robust biofilm formation. Notably, this strain persisted for three months in the toe secretion of a 58-year-old male patient with type 2 diabetes mellitus. Despite antibiotic treatment and undergoing three operations of toe amputation and skin grafting, the condition worsened, leading to the necessity of foot amputation due to progressive tissue necrosis. Phenotypic analysis showed the *S.* Thompson strain JNQH950 could form strong biofilm compared with other strains. It is suspected that bacteria are difficult to eradicate when protected within biofilms, thus acquire the enhanced ability to persist and spread in the human body or the environment (Lamas et al., 2018; Gual-De-Torrella et al., 2022).

*Salmonella* sp. has the capability to develop biofilms, and bacteria within these biofilms exhibit increased resistance to drugs, chemicals, physical and mechanical stresses, as well as evasion of host immune responses (Lamas et al., 2018). The primary constituents of *Salmonella* biofilms are curli fimbriae and cellulose (Lamas et al., 2018). Interestingly, genetic analysis revealed an intact *mrkABCDF* operon in strain JNQH950, which had been demonstrated associated with type 3 fimbriae expression, surface attachment, and biofilm formation in *K. pneumoniae* (Norman et al., 2008; Ong et al., 2009). The *mrkABCDF* operon harboring plasmid had highly conserved plasmid synteny and structure with a previously reported pOLA52 plasmid in *E.coli*, which also is an IncX1 plasmid and showed a high conjugative capability (Norman et al., 2008). In addition, the gene cluster had acquired mobility flanking by IS1 (Tn6011) compared to its original gene cluster (Norman et al., 2008). Further genetic analysis showed the *mrkABCDF* gene cluster has now spread among various enterobacterial species, but was not reported in *S. enterica* strains (**Figure 7**). In this study, although the conjugation was not performed due to the lack of selective marker, we can suspect the *mrkABCDF* loci could be mobilized through plasmid conjugation or genetic loci transposon. Taken together, to the best of our knowledge, this was the first report of *mrkABCDF* operon within *S. enterica* responsible for human infection. The emergence of such *Salmonella* strain emphasizes the urgent need for meticulous monitoring and surveillance to address potential public health implications. Nevertheless, the molecular mechanism responsible for the contribution of *mrkABCDF* to biofilm formation and enhanced antibiotic treatment resistance in *S. enterica* is yet to be elucidated.

The increasing prevalence of antibiotic resistance, particularly towards fluoroquinolones and third-generation cephalosporins, presents formidable challenges in clinical management (Fierer, 2022). Infections caused by antimicrobial-resistant *Salmonella*, especially those resistant to quinolones, pose a significant threat to human populations, leading to higher morbidity and mortality rates (Varma et al., 2005; Xia et al., 2009). Of particular concern is the emergence of nontyphoidal *Salmonella* strains that exhibit resistance to extended-spectrum cephalosporins, such as ceftazidime and ceftriaxone, which raises substantial public health concerns (Crump et al., 2015; Fakorede et al., 2023). These third-generation cephalosporins play a crucial role in managing invasive *Salmonella* infections, especially in vulnerable populations like children, for whom fluoroquinolones may not be the preferred treatment. Our study identified two MDR isolates that were resistant to AMP, CIP and TMP-SMZ, one of which exhibited resistance to third-generation cephalosporins. The presence of *bla*_CTX-M-15_ gene, conferring cephalosporin resistance, was located on a transferable IncB/O/K/Z plasmid. The plasmid carried all essential elements necessary for conjugation and, importantly, also harbored the type IV pilus system, known to significantly enhance conjugation both *in vivo* and *in vitro* (Neil et al., 2020). Consequently, the combination of fluoroquinolone and cephalosporin resistance, carried on a highly transferable plasmid in these *Salmonella* isolates, raises serious concerns about the potential dissemination of these resistance traits. Vigilant monitoring and the implementation of comprehensive strategies are urgently needed to address this pressing public health issue.

Our data also showed that *S.* Enteritidis were the most common serovars detected in our hospital, which is consistent with the findings in other studies in China (Wang et al., 2015; Wang et al., 2023). In addition, the proportion of *S.* Mbandaka was reported to be 0.25% of the total NTS collection, most of which were from non-human origin (Wang et al., 2023). Genetic analysis revealed that the *S.* Enteritidis strains in this study displayed significant genetic diversity, in line with findings from an epidemiological study in China (Wang et al., 2015). On the other hand, our analysis of *S.* Mbandaka isolates revealed a different scenario. These isolates formed a single clonal lineage, indicating a common origin. A recent study in China had reported *S.* Mbandaka could be clonally transmitted between broiler farm and slaughterhouse (Wang et al., 2021b). Further comparison with a large-scale collection of *S.* Mbandaka in China identified one closely related strain, which was isolated from human feces in Zhejiang in 2017. Given previous studies suggesting *S.* Mbandaka’s adaptation to survival in the farm environment (Hayward et al., 2016), our findings suggest that this clone in this study likely originated from a single source that persisted over 5 years in China.

In summary, our research provides the phenotypic and molecular overview of current serovar prevalence of NTS strains isolated from human origins in China. *S.* Enteritidis strains displayed significant genetic diversity while *S.* Mbandaka strains were suspected to derive from a single clonal expansion. The further spread of the *bla*_CTX-M-15_ harboring IncB/O/K/Z plasmids and spread of virulent *mrkABCDF* operon into *Salmonella* in China and other global regions should be closely monitored.

## Data availability statement

The datasets presented in this study can be found in online repositories. The names of the repository/repositories and accession number(s) can be found in the article/supplementary material.

## Ethics statement

The studies involving humans were approved by The First Affiliated Hospital of Shandong First Medical University. The studies were conducted in accordance with the local legislation and institutional requirements. Written informed consent for participation in this study was provided by the participants’ legal guardians/next of kin.

## Author contributions

WM: Conceptualization, Methodology, Supervision, Writing – original draft. XC: Data curation, Methodology, Writing – original draft. XD: Writing – original draft. XL: Methodology, Writing – original draft. KL: Visualization, Writing – original draft. YW: Software, Writing – original draft. XS: Project administration, Writing – original draft. LC: Validation, Visualization, Writing – review & editing. MH: Conceptualization, Supervision, Writing – original draft, Writing – review & editing.
